# Synthesis and Ultrahigh Pressure Compression of High-Entropy Boride (Hf_0.2_Mo_0.2_Nb_0.2_Ta_0.2_Zr_0.2_)B_2_ to 220 GPa

**DOI:** 10.3390/ma16010158

**Published:** 2022-12-24

**Authors:** Seth Iwan, Christopher Perreault, Yogesh K. Vohra

**Affiliations:** Department of Physics, University of Alabama at Birmingham, Birmingham, AL 35294, USA

**Keywords:** high pressure, high temperature, high-entropy borides, equation of state

## Abstract

The high-entropy boride (Hf_0.2_Mo_0.2_Nb_0.2_Ta_0.2_Zr_0.2_)B_2_ material was synthesized under high-pressures and high-temperatures in a large-volume Paris-Edinburgh (PE) press from a ball-milled powder mix of HfO_2_, MoO_3_, Nb_2_O_5,_ Ta_2_O_5_, ZrO_2_, carbon black, and boron carbide. The transformation process was monitored in situ by energy-dispersive x-ray diffraction with conversion starting at 1100 °C and completed by 2000 °C with the formation of a single hexagonal AlB_2_-type phase. The synthesized sample was recovered, powdered, and mixed with platinum pressure marker and studied under high pressure by angle-dispersive x-ray diffraction in a diamond anvil cell. The hexagonal AlB_2_-type phase of (Hf_0.2_Mo_0.2_Nb_0.2_Ta_0.2_Zr_0.2_)B_2_ was found to be stable up to the highest pressure of 220 GPa reached in this study (volume compression *V/V*_0_ = 0.70). The third order Birch-Murnaghan equation of state fit to the high-pressure data up to 220 GPa results in an ambient pressure unit cell volume $V_{0}=28.16\pm 0.04\;{Å}^{3},\;\mathrm{bulk}\;\mathrm{modulus}$ $K_{o}$ = 407 ± 6 GPa, pressure derivative of bulk-modulus $K_{0}^{\prime }$ = 2.73 ± 0.045 GPa. Our study indicates that this high-entropy boride (Hf_0.2_Mo_0.2_Nb_0.2_Ta_0.2_Zr_0.2_)B_2_ material is stable to ultrahigh pressures and temperatures and exhibit high bulk modulus similar to other incompressible transition metal borides like ReB_2_ and Os_2_B_3_.

## 1. Introduction

High Entropy Borides (HEBs) are a new class of ceramic materials that can withstand extreme high pressures and high temperatures without degradation in physical and/or mechanical properties. Simple transition metal borides have been extensively studied and been found to have desirable mechanical properties including high bulk modulus and high hardness [1,2,3,4]. The HEBs in general have five different metals in near equal molar ratio and are stable in a single hexagonal AlB_2_-type phase [5]. The inclusion of five or more different metallic elements results in high entropy (S) including entropy of mixing and hence minimized Gibbs free energy, G = H−TS, where H is the enthalpy and T is the temperature. The entropy of mixing term can be expressed as ΔS_mix_ = R ln(N), R being the gas constant and N the number of elements present. This entropy term in the free energy also ensures that the ambient temperature crystalline phases in high-entropy materials are also stable at high temperatures resulting in their applicability under extreme temperature and pressure conditions. The ambient pressure studies on High Entropy Alloys (HEAs), and HEBs have demonstrated that superior mechanical, magnetic and chemical properties can be tailored as compared to their constituent elements and compounds [6,7]. The previous static high-pressure studies on HEBs have so far been limited to pressures below 10 GPa [8,9] and their phase stability and equation of state measurements at pressures exceeding 100 GPa have not been explored. In addition, relatively little is known about the transformation kinetics of the boron-carbon thermal reduction process during the formation of HEB due to lack of in-situ observations. In this paper we focus on the real-time monitoring of the formation of HEB from the precursor materials under high-pressures and high-temperatures by X-ray diffraction and study the compression behavior of the synthesized HEB material under ultrahigh pressures in a diamond anvil cell.

**Figure 1:**
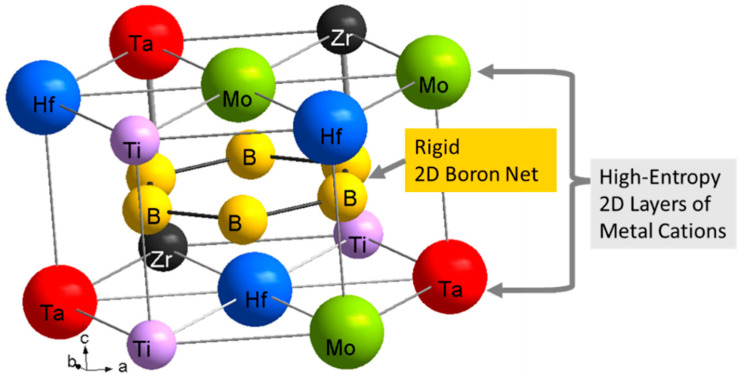
Schematic of a typical high-entropy boride crystal structure (hexagonal AlB_2_-type) showing a rigid 2D boron net and metal cations sites that are randomly occupied by the five transition metals.

The bulk-synthesis of HEB (Hf_0.2_Mo_0.2_Nb_0.2_Ta_0.2_Zr_0.2_)B_2_ material is carried out in a large-volume Paris-Edinburgh press at the Advanced Photon Source, High Pressure Collaborative Access Team (HPCAT) Beamline 16-BM-B, Argonne National Laboratory. A schematic of a typical HEB crystal structure is shown in Figure 1. (Hf_0.2_Mo_0.2_Nb_0.2_Ta_0.2_Zr_0.2_)B_2_ is synthesized under quasi-hydrostatic pressure from a mixture of transition metal oxides, Boron Carbide and Carbon black. The in-situ evolution of crystal structure was studied using the Energy Dispersive X-ray Diffraction under high pressure and high temperature (HPHT) conditions. The synthesized (Hf_0.2_Mo_0.2_Nb_0.2_Ta_0.2_Zr_0.2_)B_2_ sample was recovered from HPHT conditions and studied to 220 GPa in a diamond Anvil Cell (DAC) experiment conducted at the HPCAT beamline 16-BM-D. The ultrahigh pressure data was used to construct an isothermal equation of state (EOS) of (Hf_0.2_Mo_0.2_Nb_0.2_Ta_0.2_Zr_0.2_)B_2_ to 220 GPa at ambient temperature.

## 2. Materials and Methods

### 2.1. Mixing Procedure

(Hf_0.2_Mo_0.2_Nb_0.2_Ta_0.2_Zr_0.2_)B_2_ was synthesized under High Pressure and High temperature from an oxide precursor. The oxide precursor is prepared by mixing HfO_2_, MoO_3_, Nb_2_O_5,_ Ta_2_O_5_, ZrO_2_ with carbon black and boron carbide, all in powdered form. The metal oxide precursors are reduced in the presence of boron carbide and graphite with the formation of HEB and release of carbon monoxide as shown in Equation (1). To account for any potential loss of boron due to release of B_2_O_3_ during this boron-carbon thermal reduction process, additional B_4_C (9% by wt.) is added to ensure that enough boron is present. The purity and particle size of the precursor materials are summarized in Table 1. The oxides are mixed in a high energy ball miller (Spex 8000M) for a total of six hours to ensure small particle sizes and a uniform distribution of the oxides. The first mixing step involves the transition metal oxides along with the Carbon Black in a tungsten carbide canister with tungsten carbide medium for two hours. Before mixing for the remaining four hours, all the B_4_C is added along with enough acetone to turn powder mix into a slurry and the tungsten carbide balls are changed out for Zirconia balls. These changes are made to minimize the contamination from the tungsten carbide canister while ball milling hard materials like B_4_C. The acetone also keeps the B_4_C powder from agglomeration during the mixing process, which results in a finer powder [8,9]. During the mixing process the ball miller is cooled, hourly for 10 minutes. The sample is then dried using a vacuum oven and put through a 200-mesh sieve to ensure there are no large transition metal oxides or B_4_C particles remaining.

HfO_2_ + MoO_3_ + 1/2 Nb_2_ O_5_ + 1/2 Ta_2_O_5_ + ZrO_2_ + 19/2 C + 5/2 B_4_C → (Hf_0.2_Mo_0.2_Nb_0.2_Ta_0.2_Zr_0.2_)B_2_ + 12 CO (1)

### 2.2. High Pressure High Temperature Synthesis

The oxide precursor is taken to the Advanced Photon Source (APS) for synthesis and study of (Hf_0.2_Mo_0.2_Nb_0.2_Ta_0.2_Zr_0.2_)B_2_ under high pressure and high temperature conditions. The HPCAT beamline 16-BM-B Paris Edinburgh (PE) press is capable of applying 12,000 psi oil pressure and can reach temperatures of 2000 °C, through the use of a graphite heater element. The sample pressure is determined from the thermal equation of state of the magnesium oxide calibrant from its measured volume by X-ray diffraction. The PE press is equipped with an in situ white X-ray beam, with 10–120 KeV energy range and the beam size is 100 μm (horizontal) × 300 μm (vertical). The oxide powder is compressed into a cylindrical pellet, 2mm in height (vertical) and 1.5 mm in diameter (horizontal), by use of a tungsten carbide dies set. This pellet is then loaded into the PE press standard cell [8]. The graphite sleeve is the heater element that has been calibrated through several thermocouple experiments to control the temperature as a function of applied oil pressure and the electrical power supplied to the heater element [10]. Surrounding the graphite heater element is a MgO ring, this ring helps cell integrity to build pressure and it also serves as a pressure calibrant, using a thermal equation of state established by Kono et al. [11].

To initiate the synthesis process for HEB, the oxide Precursor is loaded into the PE press and compressed to 2000 psi and heated in 100 °C steps, collecting energy-dispersive X-ray diffraction spectra of HEB sample and MgO at each pressure and temperature step. Initially, only oxides peaks and fluorescence from the High Z material (Ta and Hf) are observed, but as temperature increase a hexagonal AlB_2_-type phase emerges. After synthesis, temperature continues to increase in 100 °C steps up to 2000 °C at which point the sample is annealed for 1 h. Once annealed, temperature is slowly ramped down and sample is left to cool for 45 min. Pressure is increased by 2000 psi and the heating cycle is repeated on the HEB sample. This is repeated up to 12,000 psi and 1900 °C reaching a measured sample pressure of 7.8 GPa.

### 2.3. Ultrahigh Pressure Diamond Anvil Cell Study

The diamond anvil cell experiment employed diamonds with 25 μm in diameter culet and a bevel angle of 8° with outer diameter of 300 μm. A sample hole of 10 μm in diameter was laser-drilled in a pre-indented rhenium gasket of 30 μm in thickness. The (Hf_0.2_Mo_0.2_Nb_0.2_Ta_0.2_Zr_0.2_)B_2_ sample synthesized in the PE press was powdered and mixed with Pt powder and placed in the 10-μm sample hole in rhenium gasket. The Pt-powder in the mixture serves as an in-situ pressure calibrant during X-ray diffraction study under high-pressures in a diamond anvil cell. The unit-cell volume of platinum pressure marker was obtained in a structural refinement [12] of X-ray diffraction data and used in the measurement of pressure using third-order Birch-Murnaghan equation of state (BM EoS) as shown in Equation (2). The *V*_0_ is the ambient pressure volume, *V* is the volume measured at high pressure, *P* is the pressure measured by the platinum pressure marker, *K*_0_ is the bulk modulus at ambient pressure, and $K_{0}^{\prime }$ is the pressure derivative *dK*_0_*/dP* of bulk modulus. The equation of state parameters derived by Yokoo et al. [13] for platinum, i.e., *K*_0_ = 276.4 GPa, $K_{0}^{\prime }$ = 5.12, and the unit cell volume *V*_0_ = 60.421 ${Å}^{3}$ were used in all pressure calculations.

*P*(*V*) = (3/2) *K*_0_ [*x*^7/3^ − *x*^5/3^] [1 + 0.75 (*K*_0_′ − 4) (*x*^2/3^ − 1)], *with x = V*_0_*/V* (2)

## 3. Results

**Figure 2:**
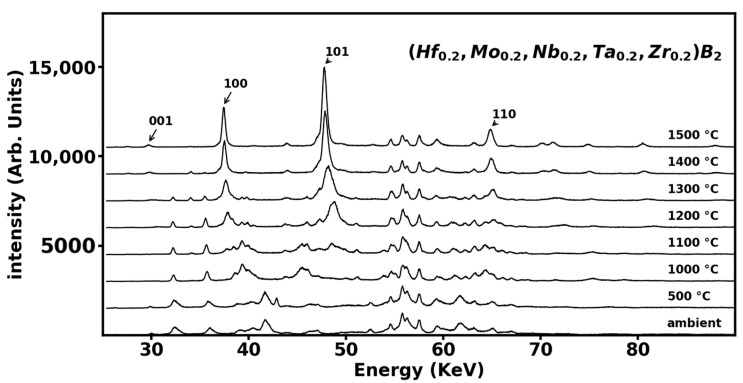
Energy-dispersive X-ray diffraction pattern of ball-milled powder mix of HfO_2_, MoO_3_, Nb_2_O_5,_ Ta_2_O_5_, ZrO_2_, carbon black, and boron carbide at various temperatures. The transformation begins at 1100 °C and by 1500 °C the (001), (100), (101) and (110) peaks of the hexagonal AlB_2_-type phase of (Hf_0.2_Mo_0.2_Nb_0.2_Ta_0.2_Zr_0.2_)B_2_ are apparent.

**Figure 3:**
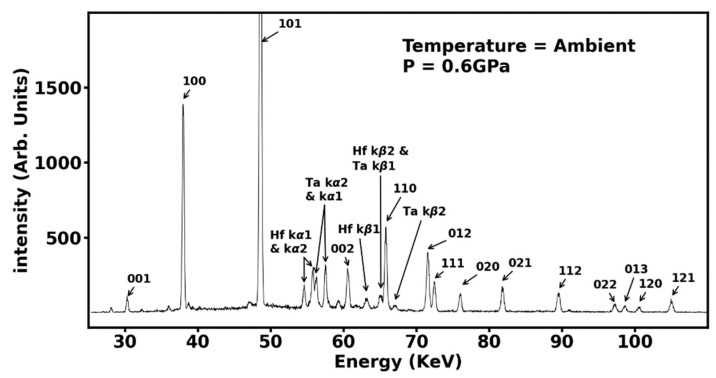
The synthesized sample of (Hf_0.2_Mo_0.2_Nb_0.2_Ta_0.2_Zr_0.2_)B_2_ at ambient temperature and at a pressure of 0.6 GPa after annealing at 2000 °C for one hour. The fourteen diffraction peaks from the hexagonal AlB_2_-type phase of (Hf_0.2_Mo_0.2_Nb_0.2_Ta_0.2_Zr_0.2_)B_2_ are indexed, indicating completion of the transformation process along with the X-ray fluorescence emission from Hf and Ta.

In Figure 2, we demonstrate in situ monitoring of the transformation process by energy-dispersive X-ray diffraction patterns recorded at various temperatures at an applied pressure of 2000 psi which correspond to a sample pressure of 0.6 GPa. At temperatures below 1100 °C, we observe the oxide diffraction peaks along with X-ray fluorescence from the constituent elements. Above 1100 °C, we begin to see gradual formation of hexagonal AlB_2_-type phase of (Hf_0.2_Mo_0.2_Nb_0.2_Ta_0.2_Zr_0.2_)B_2_ as evidenced by the appearance of (001), (100), (101), and (110) diffraction peaks of the hexagonal AlB_2_-type phase. These diffraction peaks become dominant at a higher temperature of 1500 °C (Figure 2) and the transformation was completed by 2000 °C as indicated by disappearance of diffraction peaks from all precursor materials (Figure 3). To ensure complete conversion, sample was annealed at 2000 °C for one hour and then cooled down to ambient temperature. Figure 3 shows the annealed diffraction pattern at 0.6 GPa and ambient temperature indicating complete transformation with all the observed peaks assigned to fourteen diffraction peaks of AlB_2_-type phase of (Hf_0.2_Mo_0.2_Nb_0.2_Ta_0.2_Zr_0.2_)B_2_ and the rest of the peaks assigned to X-ray fluorescence emission from Hf, and Ta. The measured lattice parameters for AlB_2_-type phase of (Hf_0.2_Mo_0.2_Nb_0.2_Ta_0.2_Zr_0.2_)B_2_ at 0.6 GPa and ambient temperature are *a* = 3.100 ± 0.001 Å and *c* = 3.360 ± 0.001 Å.

**Figure 4:**
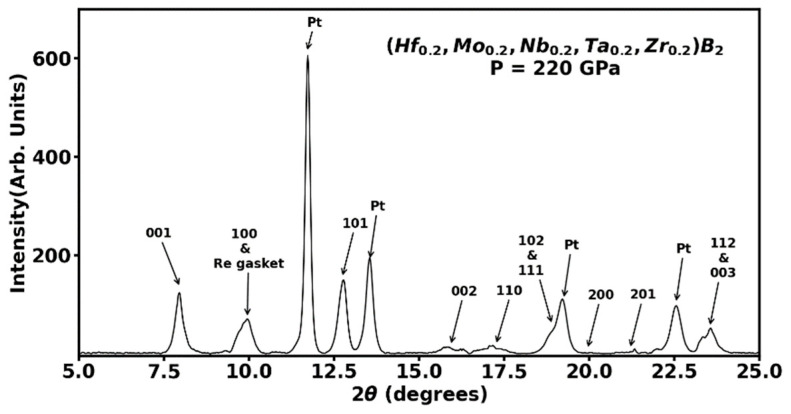
The angle dispersive X-ray diffraction pattern of (Hf_0.2_Mo_0.2_Nb_0.2_Ta_0.2_Zr_0.2_)B_2_ sample mixed with platinum (Pt) pressure marker at the highest pressure of 220 GPa. The hexagonal AlB_2_-type phase was found to be stable to 220 GPa as evidenced by the assignment of (*hkl*) Miller indices to all the observed peaks along with the face centered cubic platinum pressure standard.

The (Hf_0.2_Mo_0.2_Nb_0.2_Ta_0.2_Zr_0.2_)B_2_ sample after compression to 7.8 GPa and 2173 K was recovered, powdered, and mixed with platinum pressure maker and studied in a diamond anvil cell. In the diamond anvil cell experiment, (Hf_0.2_Mo_0.2_Nb_0.2_Ta_0.2_Zr_0.2_)B_2_ sample was compressed to 220 GPa in stepwise manner and angle dispersive X-ray diffraction data collected. Figure 4 shows the highest pressure angle-dispersive X-ray diffraction pattern at 220 GPa before decompression of sample. All the diffraction peaks in Figure 4 can be accounted for by the hexagonal AlB_2_-type phase of the sample, face centered cubic phase of platinum, and with one overlapping HEB sample peak with the rhenium gasket. This clearly indicates the wide range of stability of AlB_2_-type phase of (Hf_0.2_Mo_0.2_Nb_0.2_Ta_0.2_Zr_0.2_)B_2_ to pressures as high as 220 GPa. The fit to angle dispersive X-ray diffraction (wavelength λ = 0.4133 Å) at 220 GPa in Figure 4 results in the hexagonal phase lattice parameters *a* = 2.750 ± 0.002 Å and *c* = 2.990 ± 0.001 Å with the unit cell volume of 19.57 ± 0.02 ${Å}^{3}$ and the corresponding lattice parameter of Pt pressure marker is *a* = 3.51 ± 0.01 Å with volume compression for platinum *V/V*_0_ = 0.715. The measured compression of platinum pressure marker results in a sample pressure of 220 GPa with the use of Equation (2) as described earlier.

**Figure 5:**
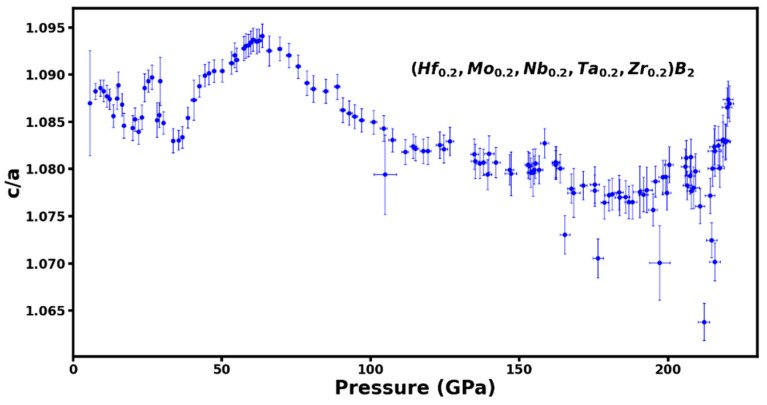
The axial (*c*/*a*) ratio for the hexagonal AlB_2_-type phase of (Hf_0.2_Mo_0.2_Nb_0.2_Ta_0.2_Zr_0.2_)B_2_ as a function of pressure to 220 GPa. The axial (*c*/*a*) ratio shows only slight variation of 2–3% over a large pressure range indicating lack of strong anisotropic compression effects.

**Figure 6:**
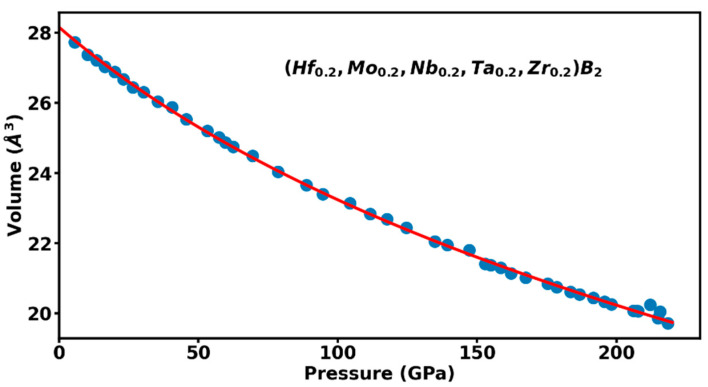
The measured Pressure-Volume relationship or equation of state of (Hf_0.2_Mo_0.2_Nb_0.2_Ta_0.2_Zr_0.2_)B_2_ to 220 GPa or volume compression *V*/*V*_0_ = 0.7. The solid curve is the third order Birch-Murnaghan equation of state fit to the experimental data and the fit parameters are described in the text.

Several transition metal borides show strong anisotropy in their compression behavior of the hexagonal lattice whereby the *c*-axis is less compressible than the *a*-axis up to the highest pressure of 241 GPa for ReB_2_ [14] and 358 GPa for Os_2_B_3_ [15]. This anisotropic compression manifests itself as an increase in the axial (*c/a*) ratio with increasing pressure and has been attributed to coulomb repulsion that occurs due to electron overlap along the c-axis making it less compressible [14]. It is of interest to investigate whether there are similar anisotropic compression effects in the hexagonal phase of high-entropy borides, e.g., (Hf_0.2_Mo_0.2_Nb_0.2_Ta_0.2_Zr_0.2_)B_2_ to the highest pressure of 220 GPa. Figure 5 shows the measured axial (*c/a*) ratio as a function of pressure where an initial slight increase in the (*c/a*) ratio is followed by a gradual decrease indicating that *c*-axis in more compressible than the *a*-axis at ultrahigh pressures exceeding 62 GPa. The overall change in the (*c/a*) ratio is within 2–3% over the 220 GPa pressure range. The measured Pressure-Volume (P-V) data or equation of state is plotted in Figure 6. The third-order Birch-Murnaghan equation of state fit to the high-pressure data up to 220 GPa results in an ambient pressure volume $V_{0}=28.16\;\pm 0.04\;{Å}^{3},\;\mathrm{bulk}\;\mathrm{modulus}$ $K_{o}$ = 407 ± 6 GPa, pressure derivative of bulk-modulus $K_{0}^{\prime }=$ 2.73 ± 0.04 GPa [16].

## 4. Discussion

Our in-situ monitoring of boron-carbon thermal reduction of ball milled oxide precursors mixed with carbon black and boron-carbide powder has indicated a sluggish transformation to HEB formation starting at 1100 °C and completing at 2000 °C. A fully dense hexagonal AlB_2_-type structure is obtained after annealing for one hour at 2000 °C. The ultrahigh pressure compression to 220 GPa in diamond anvil cell has established a wide range of stability for the AlB_2_-type phase of (Hf_0.2_Mo_0.2_Nb_0.2_Ta_0.2_Zr_0.2_)B_2_ to about 30% volume compression. Unlike other transition metal borides like ReB_2_ and Os_2_B_3_, HEB (Hf_0.2_Mo_0.2_Nb_0.2_Ta_0.2_Zr_0.2_)B_2_ does not show an increase in axial (*c/a*) ratio with increasing pressure. This implies that the strong anisotropic compression effects observed in ReB_2_ and Os_2_B_3_ where *c*-axis is less compressible than the *a*-axis are not observed in the HEB sample. In contrast, *c*-axis is slightly more compressible than the *a*-axis in HEB above 62 GPa. A detailed band-structure calculations of HEB under compression is needed to understand the changes in electronic structure and resultant charge distributions in order to explain this compression behavior. The measured bulk modulus $K_{o}$ = 407 ± 6 GPa is higher than the bulk modulus of 364 GPa value reported for ReB_2_ in a study conducted to 241 GPa [14] and comparable to bulk modulus of 397 GPa reported for Os_2_B_3_ material in a study conducted to 348 GPa [15]. It should be added that all the measurements of the bulk modulus reported above are obtained in a diamond anvil cell under nonhydrostatic compression conditions.

## 5. Conclusions

We study transformation of ball-milled powder mix of HfO_2_, MoO_3,_ Nb_2_O_5,_ Ta_2_O_5_, ZrO_2_, carbon black, and boron carbide under high-pressure and high-temperature during the formation of high-entropy boride (Hf_0.2_Mo_0.2_Nb_0.2_Ta_0.2_Zr_0.2_)B_2_. The synthesis process started at 1100 °C and a fully dense crystalline material was obtained after annealing at 2000 °C. The synthesized material was powdered and mixed with platinum and loaded in a diamond anvil cell and studied to ultrahigh pressure of 220 GPa. The hexagonal AlB_2_-type phase of (Hf_0.2_Mo_0.2_Nb_0.2_Ta_0.2_Zr_0.2_)B_2_ was found to be stable up to 30% compression in volume and the variation of axial (*c/a*) ratio shows a slight anisotropic compression with *c*-axis being more compressible than the *a*-axis above 62 GPa. The third order Birch-Murnaghan equation of state fit to the high-pressure data up to 220 GPa results in an ambient pressure volume $V_{0}=28.16\;\pm 0.039\;{Å}^{3},\;\mathrm{bulk}\;\mathrm{modulus}$ $K_{o}$ = 407 ± 6 GPa, pressure derivative of bulk-modulus $K_{0}^{\prime }$ = 2.73 ± 0.045 GPa which puts HEB on par with the category of incompressible transition metal borides like ReB_2_ and Os_2_B_3_. Further experimental characterization of the high-pressure high-temperature synthesized HEB materials by TEM/SEM/EDX analysis and theoretical band structure calculations under compression that fully account for the random distribution of the five metallic elements will lead to understanding of high pressure behavior of this new class of incompressible materials.

## Figures and Tables

**Table 1:** The purity and particle size of the starting precursor materials used in the high-pressure high-temperature synthesis of high-entropy boride.

Precursors	Purity	Particle Size
HfO_2_	99%	44 microns
MoO_3_	99.95%	<74 microns
Ta_2_O_5_	99%	<74 microns
Nb_2_O_5_	99.9%	<74 microns
ZrO_2_	99.9%	44 microns
Carbon	99.995%	2–15 microns
B_4_C	99%	44 microns

## Data Availability

All data generated or analyzed, and materials synthesized during this study are included in this published article. Additional raw data used and/or analyzed during the current study is available from the corresponding author on reasonable request.
